# The Anticancer Effects of Marine Carotenoid Fucoxanthin through Phosphatidylinositol 3-Kinase (PI3K)-AKT Signaling on Triple-Negative Breast Cancer Cells

**DOI:** 10.3390/molecules29010061

**Published:** 2023-12-21

**Authors:** Shade’ A. Ahmed, Patricia Mendonca, Samia S. Messeha, Ebenezer T. Oriaku, Karam F. A. Soliman

**Affiliations:** 1 Division of Pharmaceutical Sciences, College of Pharmacy and Pharmaceutical Sciences, Institute of Public Health, Florida A&M University, Tallahassee, FL 32307, USA; shade1.ahmed@famu.edu (S.A.A.); ebenezer.oriaku@famu.edu (E.T.O.); 2 Department of Biology, College of Science and Technology, Florida A&M University, Tallahassee, FL 32307, USA; samia.messeha@famu.edu

**Keywords:** fucoxanthin, TNBC, PI3K-AKT signaling pathway, tumor microenvironment

## Abstract

Triple-negative breast cancer (TNBC) is an aggressive subtype of breast cancer that lacks specific targets such as estrogen, progesterone, and HER2 receptors. TNBC affects one in eight women in the United States, making up 15–20% of breast cancer cases. Patients with TNBC can develop resistance to chemotherapy over time, leading to treatment failure. Therefore, finding other options like natural products is necessary for treatment. The advantages of using natural products sourced from plants as anticancer agents are that they are less toxic, more affordable, and have fewer side effects. These products can modulate several cellular processes of the tumor microenvironment, such as proliferation, migration, angiogenesis, cell cycle arrest, and apoptosis. The phosphatidyl inositol 3-kinase (PI3K)-AKT signaling pathway is an important pathway that contributes to the survival and growth of the tumor microenvironment and is associated with these cellular processes. This current study examined the anticancer effects of fucoxanthin, a marine carotenoid isolated from brown seaweed, in the MDA-MB-231 and MDA-MB-468 TNBC cell lines. The methods used in this study include a cytotoxic assay, PI3K-AKT signaling pathway PCR arrays, and Wes analysis. Fucoxanthin (6.25 µM) + TNF-α (50 ng/mL) and TNF-α (50 ng/mL) showed no significant effect on cell viability compared to the control in both MDA-MB-231 and MDA-MB-468 cells after a 24 h treatment period. PI3K-AKT signaling pathway PCR array studies showed that in TNF-α-stimulated (50 ng/mL) MDA-MB-231 and MDA-MB-468 cells, fucoxanthin (6.25 µM) modulated the mRNA expression of 12 genes, including *FOXO1*, *RASA1*, *HRAS*, *MAPK3*, *PDK2*, *IRS1*, *EIF4EBP1*, *EIF4B*, *PTK2*, *TIRAP*, *RHOA*, and *ELK1*. Additionally, fucoxanthin significantly downregulated the protein expression of IRS1, EIF4B, and ELK1 in MDA-MB-231 cells, and no change in the protein expression of EIF4B and ELK1 was shown in MDA-MB-468 cells. Fucoxanthin upregulated the protein expression of RHOA in both cell lines. The modulation of the expression of genes and proteins of the PI3K-AKT signaling pathway may elucidate fucoxanthin’s effects in cell cycle progression, apoptotic processes, migration, and proliferation, which shows that PI3K-AKT may be the possible molecular mechanism for fucoxanthin’s effects. In conclusion, the results obtained in this study elucidate fucoxanthin’s molecular mechanisms and indicate that fucoxanthin may be considered a promising candidate for breast cancer-targeted therapy.

## 1. Introduction

An estimated 297,790 women in the United States will be diagnosed with invasive breast cancer in 2023 [1]. Approximately 13% of American women will be diagnosed with breast cancer throughout their lifetime [2]. Triple-negative breast cancer (TNBC) is an aggressive and metastatic form of breast cancer that is not easily treatable due to its triple-negative status for the progesterone receptor, the estrogen receptor, and human epidermal growth factor receptor-2. TNBC is one of the most aggressive breast cancer subtypes with rapid metastasis and poor prognosis. It accounts for 10–15% of breast cancers, and its mortality rate is 40% within the first five years after diagnosis [3].

Moreover, conventional chemotherapy is not highly effective in treating TNBC [4]. since TNBC patients can develop resistance to chemotherapy over time, leading to treatment failure. Therefore, finding other options like natural products is necessary for treatment. Natural compounds that can be used as anticancer agents are less toxic and affordable, offering strategies to overcome chemoresistance and present anti-inflammatory, anti-proliferative, anti-angiogenic, and antioxidant properties [5].

Many natural products, such as carotenoids, taxanes, and flavonoids, effectively display antitumor activity and have minimal toxicity on normal healthy cells and tissues [6]. Fucoxanthin (3′-acetoxy-5, 6-epoxy-3, 5′-dihydroxy-6′, 7′-dinero-5,6,7,8,5′,6′-hexahydro-β,β-carotene-8-one) is a marine xanthophyll which contributes to more than 10% of the carotenoids isolated from brown seaweeds such as *Fucus vesiculosus* [7], *Hijikia fusiformis* [8], *Undaria pinnatifida* [9], *Petalonia binghamiae* [10], and *Laminaria japponica* [11]. 

Fucoxanthin is metabolized into fucoxanthinol by digestive enzymes in the gastrointestinal tract, such as esterase and lipase [12]. Fucoxanthinol is further metabolized into amarouciaxanthin A through dehydrogenation in the liver. Fucoxanthin is a safe carotenoid with no side effects at 0.5% *w*/*v* in human skin and 20–2000 mg/kg body weight in rodents. Toxicity studies conducted on rats for 4 weeks, with oral dosing of fucoxanthin, showed no harmful adverse effects after daily treatments. Additionally, in vivo investigations with fucoxanthinol showed no significant adverse effects [13].

Previous studies suggest that fucoxanthin exhibits several anticancer effects in various cancer cell lines. These effects include anti-proliferation, anti-migration, anti-angiogenic, cell cycle arrest, apoptotic, and tumor inhibition [14,15]. Fucoxanthin’s anticancer potential has also been demonstrated through the inhibition of metastasis and by decreased cell growth, angiogenesis, and the induction of cell cycle arrest and apoptosis, as described in our previous literature review and studies [16,17].

Fucoxanthin was found to induce cell cycle arrest and apoptosis in various cancer cell lines such as human colon carcinoma WiDr cells, hepatocarcinoma HepG2 cells, gastric cancer adenocarcinoma MGC-803 cells, and T-cell leukemia cells [18]. In prostate cancer DU145 cells, human cervical cancer HeLa cells, and breast cancer MCF-7 cells, fucoxanthin exhibited anti-proliferative effects [19,20,21]. Fucoxanthin also displayed anti-angiogenic effects in human lymphatic endothelial cells (HLEC) and was found to downregulate the expression of VEGF-C, NF-κB, phospho-AKT, and phospho-PI3K [22]. In human glioma cancer cell lines, fucoxanthin at concentrations of 25 and 50 μM was shown to decrease the expression of phosphorylated AKT and mTOR, showing that fucoxanthin inhibits the PI3K/AKT/mTOR pathway [23].

Several signaling pathways, such as phosphatidylinositol 3-kinase (PI3K), AKT, mitogen protein kinase (MAPK), and nuclear factor kappa B (NF-κB), are associated with TNBC progression. These pathways are involved in cancer cell proliferation, the cell cycle, apoptosis, migration, invasion, and cell survival [24]. The PI3K-AKT signaling pathway, in particular, performs a vital role in selecting cellular processes, including proliferation, motility, cell growth, apoptosis, angiogenesis, and tumor survival [25]. The PI3K-AKT signaling pathway is important in regulating inflammatory pathways, which can be modulated by tumor necrosis factor-α (TNF-α) [26]. TNF-α is an important inflammatory cytokine that is a major contributor to tumor progression and is secreted into the tumor microenvironment [27]. The tumor microenvironment in TNBC is essential in tumor progression and inflammatory promotion. It is also involved in all stages of breast cancer development, affecting tumor growth, proliferation, survival, and recurrence [28]. TNF-α binds to TNF-receptor 1 (TNFR1) and can activate NF-κB, which promotes cyclin D1 and cell proliferation, to induce the PI3K-AKT signaling pathway [29]. TNF-α has also been found to increase CXCL10 expression, which plays a role in cell growth, apoptosis, and angiogenesis effects through the PI3K-AKT pathway [30,31].

The molecular genetic profiles of Caucasian and African American TNBC cell line populations are poorly characterized [32]. The genomic profiles of each TNBC cell line have been shown to differ, showing altered gene expression of the *AKT*, *TP53*, and *NFB1* pathways amongst Caucasian and African American TNBC cell lines [33]. A data study of TCGA with 90 Caucasian and 52 African American patients revealed that African American (28.42%) patients had a higher TNBC incidence than Caucasian American (11.89%) patients. A MAPK signaling gene set showed upregulation of *GADD45C*, *MAPK12*, *TRADD*, *NTRK1*, and *FGFR2* genes in African American patients compared to Caucasian patients, which could contribute to African American TNBC patients having the worst prognosis [34].

To elucidate fucoxanthin’s molecular mechanisms in proliferation, migration, angiogenesis, and cell cycle arrest in these genetically different TNBC cell lines, the current study aimed to examine fucoxanthin’s modulatory effects through TNF-α on the PI3K-AKT signaling pathway in the MDA-MB-231 and MDA-MB-468 TNBC cell lines.

## 2. Results

### 2.1. Fucoxanthin Inhibited Cell Viability in TNF-α-Stimulated MDA-MB-231 and MDA-MB-468 Cell Lines

**Figure 1:**
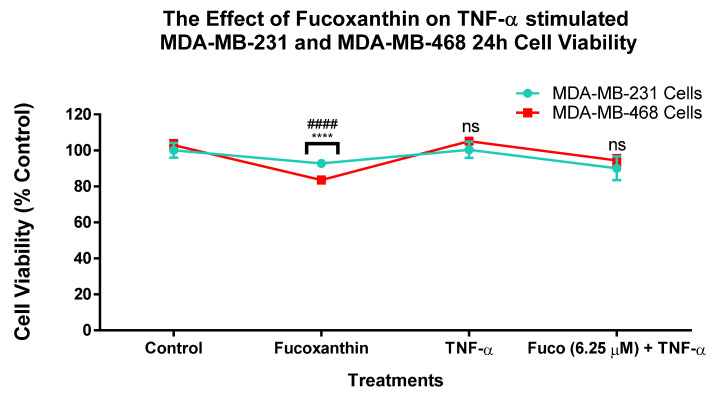
The effect of fucoxanthin on cell viability in MDA-MB-231 and MDA-MB-468 TNBC cells. The effect of fucoxanthin was tested at 6.25 µM on cell viability. The control cells were treated with DMSO (<0.1%). Each experiment was performed 3 times with n = 5. The cytotoxic effect was measured at 24 h of treatment. The data are presented as the mean ± SEM. Statistically significant differences between control and treatments were evaluated by a one-way ANOVA, followed by Dunnett’s multiple comparison tests. **** *p* < 0.0001 (MDA-MB-231 cells), #### *p* < 0.0001 (MDA-MB-468 cells), ns = *p* > 0.05.

The effect of fucoxanthin on cell viability in TNBC was investigated in the MDA-MB-231 and MDA-MB-468 cell lines after 24 h of treatment (Figure 1). The results show that both cell lines produced cell viability values over 75% in all the different treatment groups (control, fucoxanthin (6.25 µM), TNF-α, and fucoxanthin (6.25 µM) + TNF-α (50 ng/mL)).

### 2.2. Fucoxanthin Modulation of the PI3K-AKT Signaling Pathway in MDA-MB-231 and MDA-MB-468 TNBC Cells

**Figure 2:**
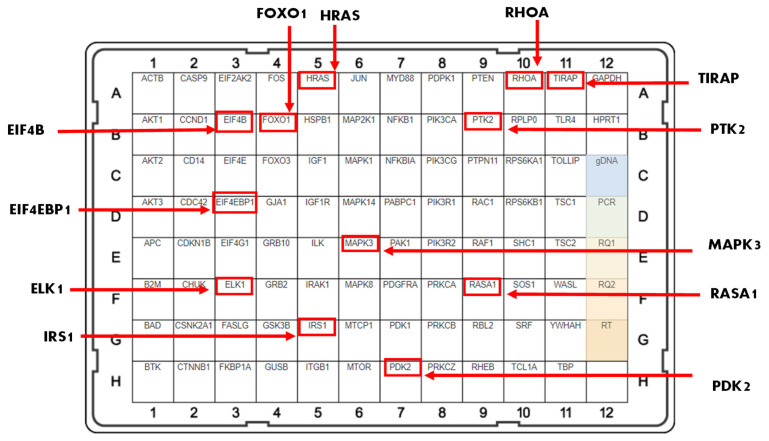
The mRNA expression of 12 genes in the PI3K-AKT signaling pathway was modulated (indicated by red boxes) by fucoxanthin (6.25 µM) + TNF-α (50 ng/mL) compared to TNF-α treatment in the MDA-MB-231 and MDA-MB-468 cell lines.

**Figure 3:**
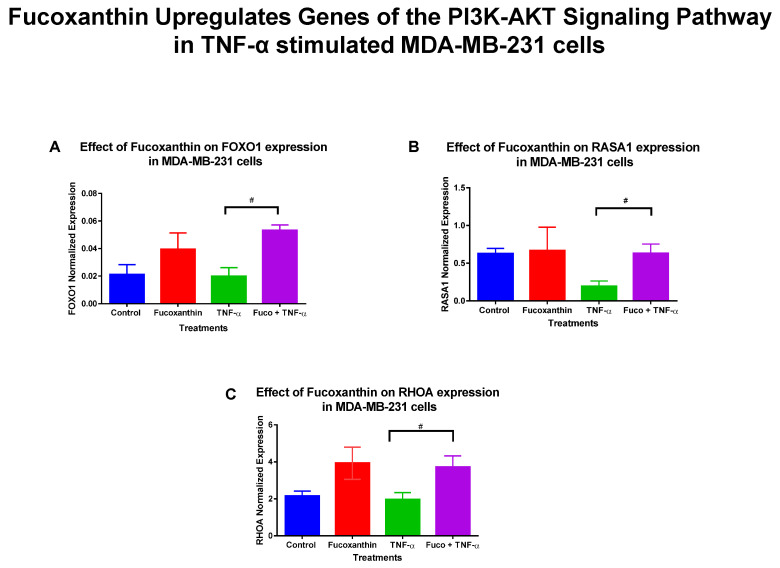
Fucoxanthin upregulated the mRNA expression of PI3K-AKT signaling pathway genes in TNF-α-stimulated MDA-MB-231 cells. Fucoxanthin upregulated the expression of 3 genes in the TNF-α-stimulated PI3K-AKT signaling pathway in MDA-MB-231 cells. These genes include forkhead box 1 (*FOXO1*) (**A**), Ras P21 protein activator (*RASA1*) (**B**), and Rho family protein A (*RHOA*) (**C**). Each data point represents the mean ± SEM, representing 4 treatments: control (cells + DMSO), fucoxanthin (6.25 µM), TNF-α (50 ng/mL), and fucoxanthin (6.25 µM) + TNF-α (50 ng/mL). A *t*-test was used to evaluate statistically significant differences between TNF-α and fucoxanthin + TNF-α. ns = *p* > 0.05, # *p* < 0.05.

**Figure 4:**
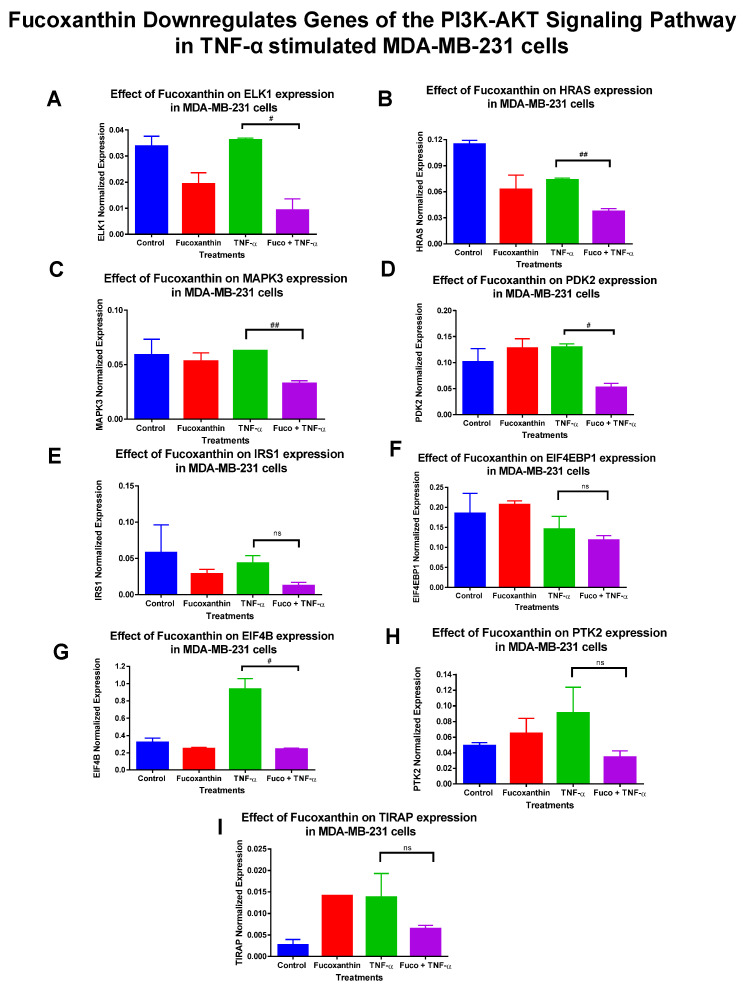
Fucoxanthin downregulated the mRNA expression of PI3K-AKT signaling pathway genes in TNF-α-stimulated MDA-MB-231 cells. The data display the effects of fucoxanthin on the *mRNA* gene expression of genes associated with the PI3K-AKT signaling pathway. Fucoxanthin downregulated the expression of 10 genes in TNF-α-stimulated MDA-MB-231 cells. These genes include ETS transcription factor (*ELK1*) (**A**), HRas Proto-Oncogene, GTPase (*HRAS*) (**B**), mitogen-activated protein kinase 3 (*MAPK3*) (**C**), pyruvate dehydrogenase kinase 2 (*PDK2*) (**D**), insulin receptor substrate 1(*IRS1*) (**E**), eukaryotic transition initiation factor 4E-binding protein 1 (*EIF4EBP1*) (**F**), eukaryotic translation initiation factor 4b (*EIF4B*) (**G**), protein tyrosine kinase 2 (*PTK2*) (**H**), and TIR domain-containing adaptor protein (*TIRAP*) (**I**). Each datapoint represents the mean ± SEM, representing 4 treatments: control (cells + DMSO), fucoxanthin (6.25 µM), TNF-α (50 ng/mL), and fucoxanthin (6.25 µM) + TNF-α (50 ng/mL). A *t*-test evaluated statistically significant differences between TNF-α and fucoxanthin + TNF-α (#). ns = *p* > 0.05, # *p* < 0.05, ## *p* < 0.01.

**Figure 5:**
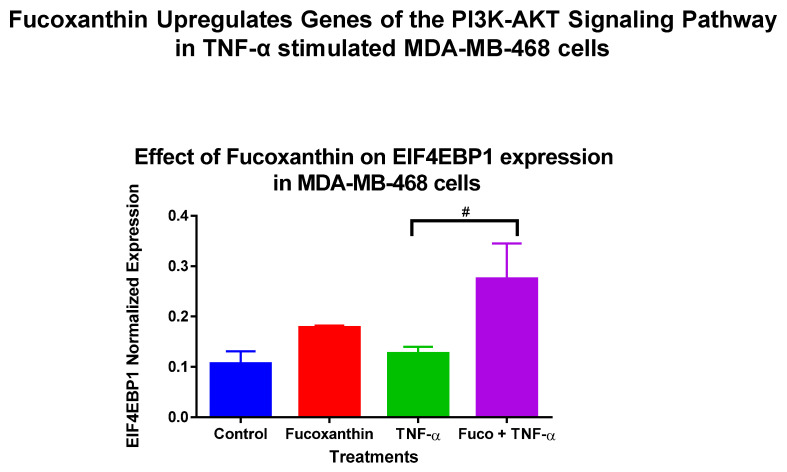
Fucoxanthin upregulated the mRNA expression of the PI3K-AKT signaling pathway EIF4EBP1 in TNF-α-stimulated MDA-MB-468 cells. The data display the effects of fucoxanthin on mRNA gene expression in the PI3K-AKT signaling pathway of TNF-α-stimulated MDA-MB-468 cells. Fucoxanthin upregulated the expression of eukaryotic transition initiation factor 4E-binding protein 1 (*EIF4EBP1*) in TNF-α-stimulated MDA-MB-468 cells. Each data point represents the mean ± SEM, representing 4 treatments: control (cells + DMSO), fucoxanthin (6.25 µM), TNF-α (50 ng/mL), and fucoxanthin (6.25 µM) + TNF-α (50 ng/mL). A *t*-test evaluated statistically significant differences between TNF-α and fucoxanthin + TNF-α (#). # *p* < 0.05.

**Figure 6:**
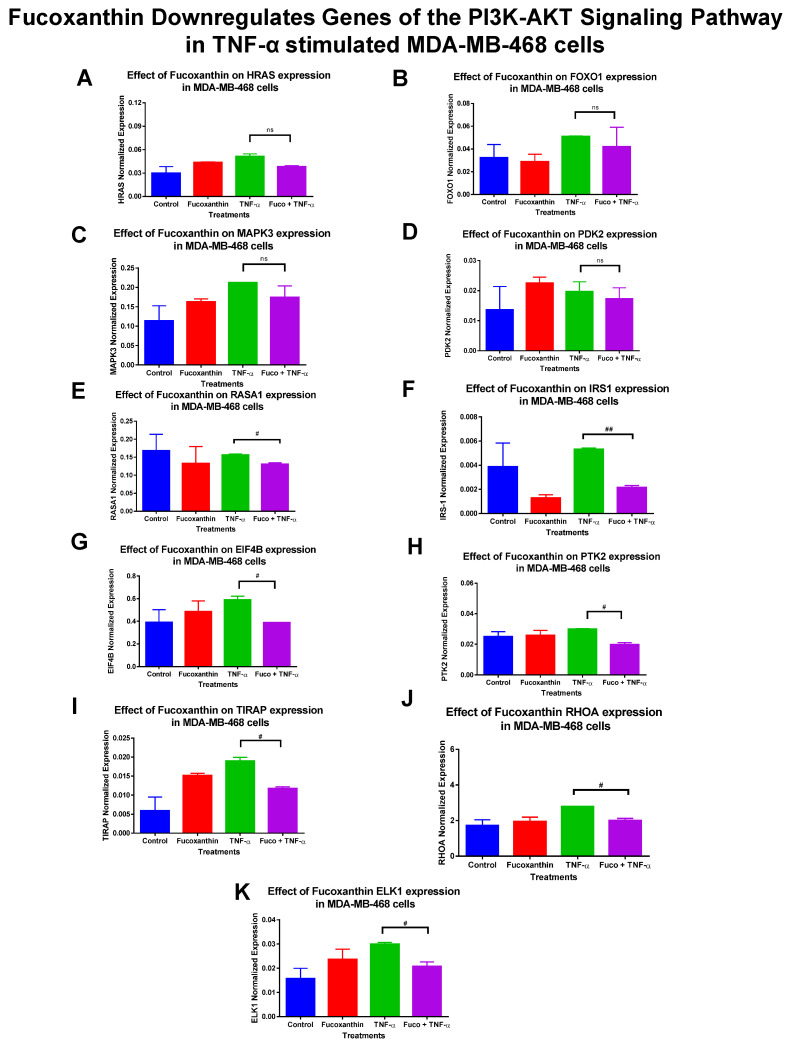
Fucoxanthin downregulated the mRNA expression of PI3K-AKT signaling pathway genes in TNF-α-stimulated MDA-MB-468 cells. The data display the effects of fucoxanthin on *mRNA* gene expression in the PI3K-AKT signaling pathway of TNF-α-stimulated MDA-MB-468 cells. Fucoxanthin upregulated the expression of 11 genes in TNF-α-stimulated MDA-MB-468 cells. These genes include HRas Proto-Oncogene, GTPase (*HRAS*) (**A**) forkhead box 1 (*FOXO1*) (**B**), mitogen-activated protein kinase 3 (*MAPK3*) (**C**), pyruvate dehydrogenase kinase 2 (*PDK2*) (**D**), Ras P21 protein activator (*RASA1*) (**E**), insulin receptor substrate 1(*IRS1*) (**F**), eukaryotic translation initiation factor 4b (*EIF4B*) (**G**), protein tyrosine kinase 2 (*PTK2*) (**H**), TIR domain-containing adaptor protein (*TIRAP*) (**I**), Rho family protein A (*RHOA*) (**J**), and ETS transcription factor (*ELK1*) (**K**). Each data point represents the mean ± SEM, representing 4 treatments: control (cells + DMSO), fucoxanthin (6.25 µM), TNF-α (50 ng/mL), and fucoxanthin (6.25 µM) + TNF-α (50 ng/mL). A *t*-test evaluated statistically significant differences between TNF-α and fucoxanthin + TNF-α (#). ns = *p* > 0.05, # *p* < 0.05, ## *p* < 0.01.

The mRNA expression of 89 genes mediating the PI3K-AKT signaling pathway was investigated in the MDA-MB-231 and MDA-MB-468 cell lines. Both cell lines were exposed to treatment groups consisting of control (cells + DMSO), fucoxanthin (6.25 µM), TNF-α (50 ng/mL), and fucoxanthin (6.25 µM) + TNF-α (50 ng/mL). The expression of 12 genes was modulated by fucoxanthin in the MDA-MB-231 and MDA-MB-468 cell lines (Figure 2). In MDA-MB-231 cells, the expression of three tumor suppressor genes was significantly upregulated by fucoxanthin compared to TNF-α alone (Figure 3). These genes include forkhead box 1 (*FOXO1*) (Figure 3A), Rho family protein A (*RHOA*) (Figure 3B), and Ras P21 protein activator (*RASA1*) (Figure 3C). In MDA-MB-231 cells, the expression of nine genes was significantly downregulated by fucoxanthin compared to TNF-α treatment only (Figure 4). These genes include ETS transcription factor (*ELK1*) (Figure 4A), HRas Proto-Oncogene, GTPase (*HRAS*) (Figure 4B), mitogen-activated protein kinase-3 (*MAPK3*) (Figure 4C), pyruvate dehydrogenase kinase 2 (*PDK2*) (Figure 4D), insulin receptor substrate 1 (*IRS1*) (Figure 4E), eukaryotic transition initiation factor 4E-binding protein 1 (*EIF4EBP1*) (Figure 4F), eukaryotic translation initiation factor 4b (*EIF4B*) (Figure 4G), protein tyrosine kinase 2 (*PTK2*) (Figure 4H), and TIR domain-containing adaptor protein (*TIRAP*) (Figure 4I). In MDA-MB-468 cells, the expression of *EIF4EBP1* (Figure 5) was significantly upregulated by fucoxanthin compared to TNF-α alone. Also, in the MDA-MB-468 cells, the expression of 11 genes was significantly downregulated compared to TNF-α alone (Figure 6). These genes include *HRAS* (Figure 6A), *FOXO1* (Figure 6B), *MAPK3* (Figure 6C), *PDK2* (Figure 6D), *RASA1* (Figure 6E), *IRS1* (Figure 6F), *EIF4B* (Figure 6G), *PTK2* (Figure 6H), *TIRAP* (Figure 6I), *RHOA* (Figure 6J), and *ELK1* (Figure 6K). In MDA-MB-231 cells, the fold changes (FCs) for significantly upregulated genes were shown to be *FOXO1* (+2.66), *RASA1* (+3.24), and *RHOA* (+1.89). The FCs for downregulated genes in MDA-MB-231 cells were *HRAS* (−1.97), *MAPK3* (−1.90), *PDK2* (−2.46), *IRS1* (−3.51), *EIF4EBP1* (−1.23), *EIF4B* (−3.83), *PTK2* (−2.64), *TIRAP* (−2.14), and *ELK1* (−3.91) (Table 1). In MDA-MB-468 cells, the FC for the significantly upregulated gene was *EIF4EBP1* (+2.16). The FCs for MDA-MB-468 downregulated genes were shown to be *FOXO1* (−1.21), *HRAS* (−1.34), *MAPK3* (−1.22), *PDK2* (−1.14), *RASA1* (−1.19), *IRS1* (−1.49), *EIF4B* (−1.51), *PTK2* (−1.51), *TIRAP* (−1.61), *RHOA* (−1.38), and *ELK1* (−1.44) (Table 2).

### 2.3. Fucoxanthin Modulated PI3K-AKT Signaling Pathway Protein Expression in MDA-MB-231 and MDA-MB-468 Cells

**Figure 7:**
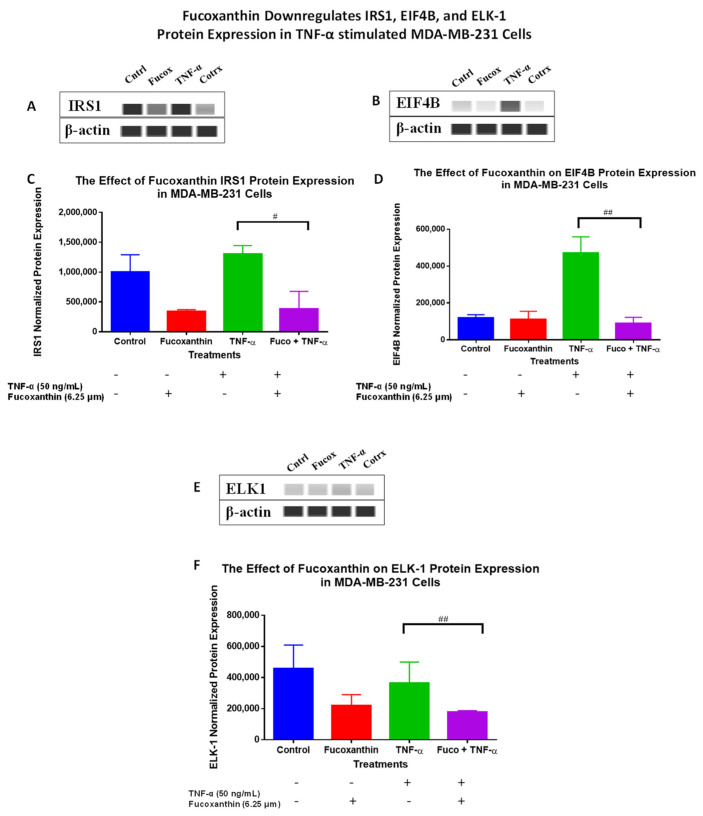
The data display the effects of fucoxanthin on IRS1 (**A**,**C**), EIF4B (**B**,**D**), and ELK1 (**E**,**F**) protein expression with quantitative normalized protein expression in TNF-α-stimulated MDA-MB-231 cells. Immunodetection of bands was achieved via Wes analysis from ProteinSimple www.bio-techne.com/brands/proteinsimple (accessed on 6 May 2023). Each data point represents the mean ± SEM, representing 4 treatments: control (cells + DMSO), fucoxanthin (6.25 µM), TNF-α (50 ng/mL), and fucoxanthin (6.25 µM) + TNF-α (50 ng/mL). A *t*-test was used to evaluate statistically significant differences between TNF-α and fucoxanthin + TNF-α (#). # *p* < 0.05, ## *p* < 0.01.

**Figure 8:**
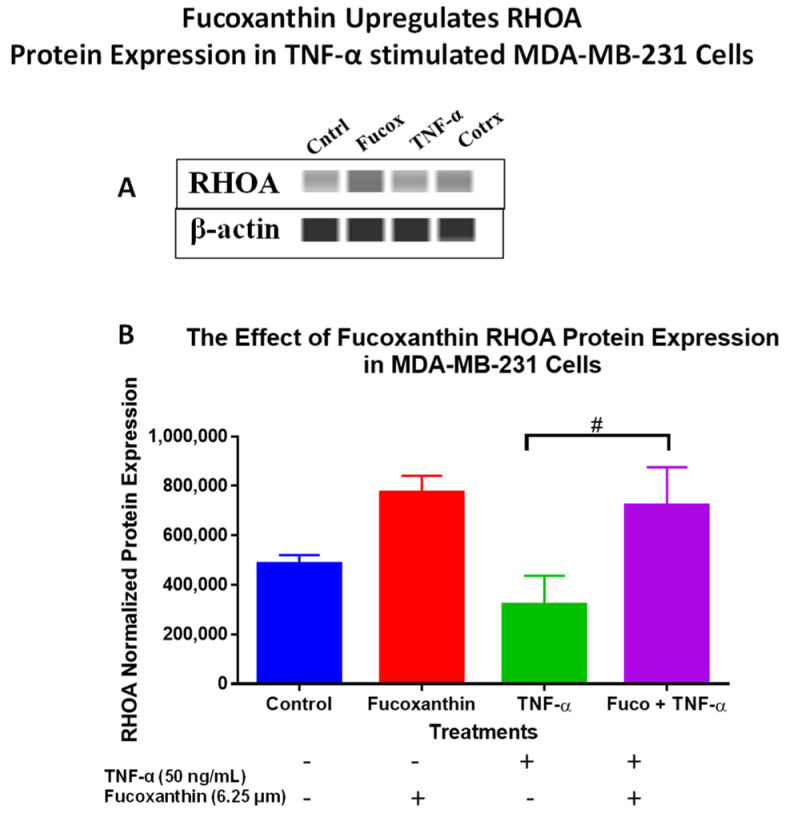
The data display the effects of fucoxanthin on RHOA (**A**,**B**) protein expression and quantitative normalized protein expression in TNF-α-stimulated MDA-MB-231 cells. Immunodetection of bands was achieved via Wes analysis using ProteinSimple. Each data point represents the mean ± SEM, representing 4 treatments: control (cells + DMSO), fucoxanthin (6.25 µM), TNF-α (50 ng/mL), and fucoxanthin (6.25 µM) + TNF-α (50 ng/mL). A *t*-test was used to evaluate statistically significant differences between TNF-α and fucoxanthin + TNF-α (#). # *p* < 0.05.

**Figure 9:**
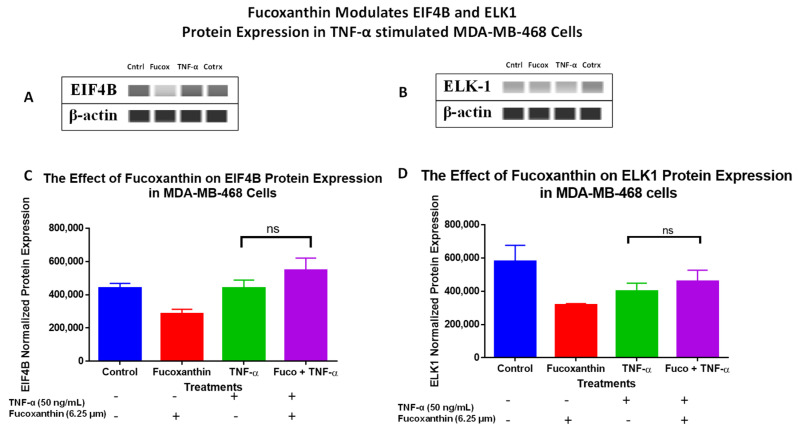
The data display the effects of fucoxanthin on EIF4B (**A**,**C**) and ELK1 (**B**,**D**) protein expression and quantitative normalized expression in TNF-α-stimulated MDA-MB-468 cells. Immunodetection of bands was achieved via Wes analysis using ProteinSimple. Each datapoint represents the mean ± SEM, representing 4 treatments: control (cells + DMSO), fucoxanthin (6.25 µM), TNF-α (50 ng/mL), and fucoxanthin (6.25 µM) + TNF-α (50 ng/mL). A *t*-test was used to evaluate statistically significant differences between TNF-α and fucoxanthin + TNF-α (#). ns = *p* > 0.05.

**Figure 10:**
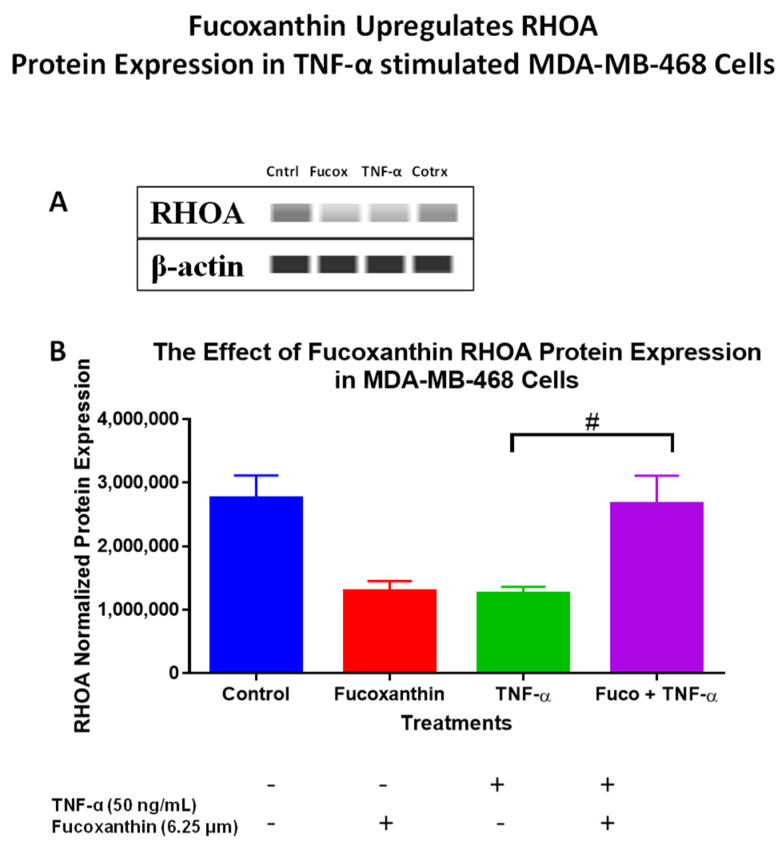
The data display the effects of fucoxanthin on RHOA (**A**,**B**) protein expression and quantitative normalized protein expression in TNF-α-stimulated MDA-MB-468 cells. Immunodetection of bands was achieved via Wes analysis using ProteinSimple. Each datapoint represents the mean ± SEM, representing 4 treatments: control (cells + DMSO), fucoxanthin (6.25 µM), TNF-α (50 ng/mL), and fucoxanthin (6.25 µM) + TNF-α (50 ng/mL). A *t*-test evaluated statistically significant differences between TNF-α and fucoxanthin + TNF-α (#). # *p* < 0.05.

The protein levels of the PI3K-AKT PCR array results were verified using Wes analysis, which was performed using total proteins and specific antibodies for IRS-1 (Figure 7A,C), EIF4B (Figure 7B,D), ELK-1 (Figure 7E,F), and RHOA (Figure 8A,B) in MDA-MB-231 cells, and specific antibodies for EIF4B (Figure 9A,C), ELK1 (Figure 9B,D), and RHOA (Figure 10A,B) in MDA-MB-468 cells. The stimulation of cells with TNF-α upregulated IRS-1, EIF4B, and ELK-1 and downregulated RHOA protein expression in MDA-MB-231 cells. However, co-treatment with fucoxanthin + TNF-α downregulated IRS-1, EIF4B, and ELK-1 and upregulated RHOA protein expression in MDA-MB-231 cells (Figure 7 and Figure 8). In MDA-MB-468 cells, stimulation with TNF-α downregulated RHOA, and no change was shown for ELK-1 and EIF4B protein expression. Co-treatment (fucoxanthin + TNF-α) upregulated RHOA protein expression, and no expression change was shown for ELK-1 and EIF4B protein expression in MDA-MB-468 cells (Figure 9 and Figure 10).

### 2.4. Activation of the PI3K-AKT Signaling Pathway through Stimulation of TNF-α Using KEGG Analysis Database

**Figure 11:**
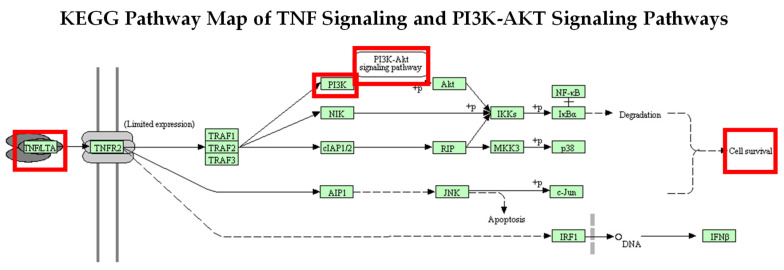
KEGG TNF-α signaling and its association with the PI3K-AKT signaling pathway. The KEGG pathway map displays the PI3K-AKT pathway activated by TNF signaling. TNF binds to TNF receptor 2 (TNFR2) and mediates intracellular signaling, which activates many genes, leading to cell survival and apoptosis. TNFR2 leads to the stimulation of PI3K, which then activates AKT and cell survival. Red boxes indicate TNF activation of the PI3K-AKT signaling pathway.

The KEGG (Kyoto Encyclopedia of Genes and Genomes) database was used to show PI3K-AKT signaling activation in this investigation. The KEGG pathway displays PI3K-AKT pathway activation by TNF signaling (Figure 11), where TNF binds to the TNF receptor 2 (TNFR2) and mediates intracellular signaling, activating many genes that induce cell survival and apoptosis. TNFR2 stimulates PI3K, which then activates AKT and induces tumor cell survival [47]. 

### 2.5. STRING, Functional Enrichment Analysis of Fucoxanthin’s Effects on Proteins of the PI3K-AKT Signaling Pathway 

**Figure 12:**
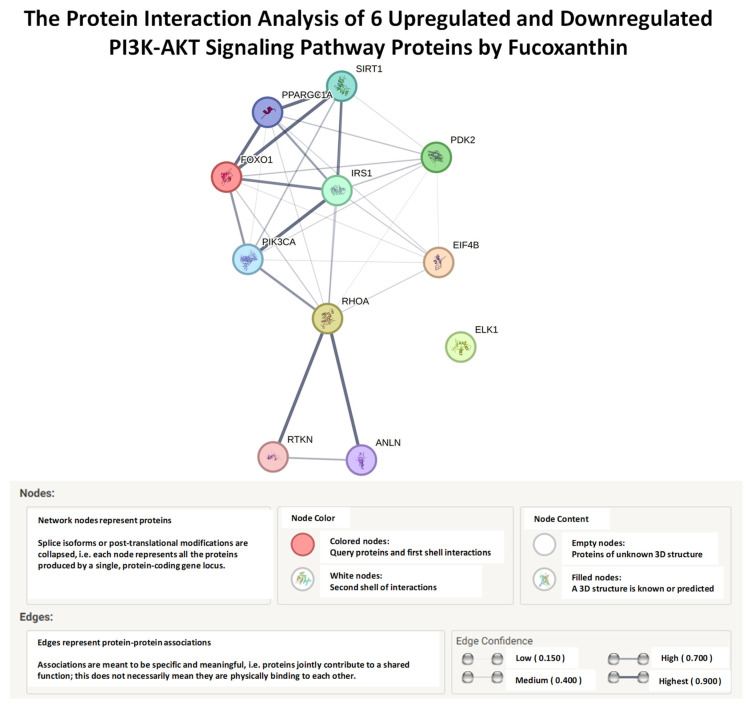
STRING, functional enrichment analysis of fucoxanthin’s effects on 6 proteins of the PI3K-AKT signaling pathway in TNF-α-stimulated MDA-MB-231 and MDA-MB-468 cells. STRING protein–protein interaction network functional enrichment analysis of PI3K-AKT signaling pathway in TNF-α-stimulated MDA-MB-231 and MDA-MB-468 cells. The network included 6 proteins with predicted functional partners, including FOXO1, RHOA, ELK1, EIF4B, IRS1, and PDK2. The legend displays the node network, color and content, and edge protein–protein associations and confidence.

Another bioinformatics tool used in this study was the STRING (search tool for retrieving interacting genes/proteins). It is a proteomic database that contains computational prediction methods, experimental data, and text collections that allow for the study of interactions of genes and proteins. The STRING functional enrichment analysis database was used to integrate and study the protein–protein analysis of six proteins of the PI3K-AKT signaling pathway, including FOXO1, PDK2, IRS1, EIF4B, RHOA, and ELK1 [48]. The network displayed the direct interaction between FOXO1, PDK2, IRS1, EIF4B, and RHOA with predicted functional partner proteins (Figure 12). The phosphatidylinositol-4, 5-bisphosphate 3 kinase (PIK3CA) gene encodes subunit p110α, which plays a role in activating PI3K-AKT signaling [49]. The interaction of FOXO1, PDK2, IRS1, EIF4B, and RHOA was also shown with the predicted functional partner, PIK3CA. However, ELK1 had no interactions with the other proteins or the associated predicted functional partner proteins. Identifying proteins that are close to neighboring proteins aids in the identification of similar biological functions within a protein network. The thickness of the edges showed protein–protein association confidence levels from low to highest (0.150–0.900). The analysis showed a high confidence interaction between FOXO1 and IRS1, a medium confidence interaction between PDK2 and IRS1 and between PDK2 and FOXO1, and a low confidence interaction between RHOA and IRS1, RHOA and FOXO1, RHOA and PDK2, and EIF4B and all proteins (Table 3). The PPI enrichment value shows the significance of interactions between all proteins. The lower the significance value, the lower the amounts of protein interactions. 

## 3. Discussion

Several natural products have been studied for their potential to treat or prevent breast cancer [50]. Specifically, marine carotenoids were investigated for their efficacy, potency, and low toxicity against breast cancer. Carotenoids target signaling pathways such as the PI3K-AKT, MAPK, and NF-κβ signaling pathways [51]. Fucoxanthin is a marine carotenoid isolated from brown seaweed and has many anticancer effects, such as anti-proliferative, anti-metastasis, cell cycle growth arrest, and apoptotic effects [52]. The PI3K-AKT signaling pathway mediates several of these cellular processes, contributing to the progression of tumors [53]. The current study examined fucoxanthin’s inductive and modulatory effects on the PI3K-AKT signaling pathway in MDA-MB-231 and MDA-MB-468 cells. 

Normal breast cells have an equilibrium between proliferation, cell cycle arrest, and apoptosis to maintain homeostasis [54]. Both apoptosis and cellular proliferation are linked by cell cycle regulators and apoptotic stimulation, which affect both processes [55]. The apoptotic process occurs when cell cycle checkpoints are interrupted, and the involved proteins cause cellular death, cell cycle arrest, or cell proliferation, thereby contributing to tumor progression. The PI3K-AKT signaling pathway has been associated with many proteins contributing to the induction of apoptosis [56], and in breast cancer cells, it can be triggered by inflammatory cytokines like TNF-α, which can induce uncontrolled cell death and disrupt the balance of anti-apoptotic and pro-apoptotic pathways [57]. The PI3K-AKT signaling pathway has been found to favor the cell cycle and regulate apoptosis. The ability to induce cell cycle arrest and inhibit cell proliferation has also been associated with inhibiting the PI3K-AKT signaling pathway [58]. It has been reported that the PI3K-AKT signaling pathway is also associated with inflammation during breast cancer progression [59].

The PI3K-AKT signaling pathway is a functional and critical pathway that consists of several genes that interconnect and are involved in cellular processes such as cell survival, cell proliferation, cell growth, cell motility, cell cycle arrest, and apoptosis in TNBC [60,61]. It has been confirmed that exposure to the inflammatory cytokine TNF-α can activate the PI3K-AKT signaling pathway in TNBC cell lines [62]. Furthermore, studies have shown that TNF-α stimulates NF-κB, J.N.K., and the PI3K-AKT signaling pathway in human breast cancer cells [63]. Several genes, such as *MAPK*, phosphatase and tensin homolog (*PTEN*), forkhead box (*FOXO*), caspase 3 (*CASP3*), x-linked inhibitor of apoptosis (*XIAP*), focal adhesion kinase (*FAK*), and nuclear factor-kappa-B (*NF-κB*), are involved in the PI3K-AKT signaling pathway [64]. Meanwhile, PI3K is an enzyme that plays a crucial role in regulating cellular processes such as cell growth, survival, and metabolism. When PI3K is phosphorylated (phospho-PI3K), it becomes activated and initiates a cascade of events that can promote cell proliferation and survival [65]. Fucoxanthin has been shown to act on the PI3K-AKT signaling pathway, thereby inducing cell cycle arrest and apoptosis and inhibiting proliferation, angiogenesis, and migration [66,67].Through the downregulation of PI3K/AKT/mTOR signaling, fucoxanthin has been proven to inhibit proliferation and stimulate apoptosis in ovarian cancer cells [68]. Fucoxanthin has also been shown to inhibit PI3K-AKT signaling by suppressing cell motility, migration, invasion, adhesion, and survival through the downregulation of p-PI3K, p-AKT, AKT1/2/3, and the focal adhesion proteins FAK/paxillin in MCF-7 cells and mouse lung tumors [69]. Studies showed that phospho-PI3K and phospho-Akt protein expressions were decreased after treatment with fucoxanthin in human lymphatic endothelial cells (HLEC). These results also showed that fucoxanthin inhibits tube migration and the formation of HLEC through suppression of the PI3K-AKT signaling pathway [70]. In glioma cells, fucoxanthin has been shown to cause the inhibition of PI3K-AKT and MAPKs in a time-dependent manner [71]. In vivo studies show that fucoxanthin inhibits PI3K/AKT/mTOR in BALB/C mice injected with U251 and glioma U87 cells [72]. Fucoxanthin has also been shown to inactivate the PI3K-AKT signaling pathway, thereby preventing metastasis in MCF-7 breast cancer cells [73]. Fucoxanthin has also been shown to inhibit PI3K-AKT signaling in several in vitro models; however, no effects of fucoxanthin on PI3K-AKT signaling have been demonstrated in MDA-MB-468 cells [74].

The present study used a PI3K-AKT signaling PCR array to identify the expression of 89 genes to attain an all-inclusive insight into signaling pathway molecular gene profiles. Through the stimulation of TNF-α, our results show that the mRNA expression of 12 genes was modulated by fucoxanthin in the MDA-MB-231 and MDA-MB-468 cell lines. Fucoxanthin was found to downregulate the expression of several genes in the PI3K-AKT signaling pathway in both the TNF-α-stimulated MDA-MB-231 and MDA-MB-468 cell lines, including *HRAS*, *MAPK3*, *PDK2*, *IRS1*, *EIF4B*, *PTK2*, *TIRAP*, and *ELK1*. Many of these genes induce several processes, such as cell cycle progression, apoptosis, cellular adhesion, mitosis, cell motility, and cytoskeleton remodeling [75,76]

Several targets of AKT signaling, such as insulin receptor substrate-1 (IRS1) and MAPK3, are involved in protein synthesis, leading to cell growth and cell cycle progression [77]. MAPKs play a role in inflammation, proliferation, apoptosis, differentiation, progression, and the development of breast cancer [78]. IRS1 is a mediator of the PI3K-AKT signaling pathway known to promote tumorigeneses. IRS proteins have been found to play an important role in breast cancer survival by regulating motility, proliferation, and cell survival [79]. IRS1 has been identified to promote PTK2 (protein tyrosine kinase 2), also known as FAK (focal adhesion kinase). PTK2 or FAK has been shown to assist in and stimulate tumor cell invasiveness. FAK is linked to inducing cell features such as cell adhesion and cellular migration, leading to metastasis and cellular invasion, and the overexpression of FAK leads to the inhibition of apoptosis [80]. Cancer cells have been found to avoid apoptosis and choose cellular pathways associated with proliferation, motility, adhesion, and migration, where the overexpression of PTK2 and IRS1 proteins plays a role in survival. Together, IRS1 and PTK2 may provide an explanation as to why MDA-MB-468 cells evaded apoptosis [81].

Harvey rat viral oncogene homolog (HRAS) is a member of the RAS family, which are GTP-binding proteins and play a crucial role in the regulation of adhesion, cytoskeleton rearrangements, proliferation, cell survival, and cell motility [82]. When activated, Ras proteins act as a cellular switch that forms an active GTP-bound form of RAS, which activates various cellular processes [83]. In breast epithelial cells, activated HRAS induces invasion through the recruitment of p38. FAK is required for PI3K-dependent breast tumorigenesis and activated Ras [84]. Pyruvate dehydrogenase kinase 2 (PDK2) is associated with proliferation, anti-apoptosis, tumor aggressiveness, and therapy resistance [85]. PDK2 has a co-responsibility with PDK1 to phosphorylate and activate AKT [86]. AKT binds to PIP3 at the plasma membrane and becomes phosphorylated by PDK1 and PDK2 [87]. PDK1 carries out partial activation of AKT, and full activation is carried out by PDK2, which acts to gain control of AKT and regulates its activity [88].

Cancer cells have a high demand for proteins that sustain an increased metabolic rate and proliferation for cancer progression. Eukaryotic translation initiation factor 4B (EIF4B) is regulated by both the Ras-MAPK and PI3K/mTOR proto-oncogenic signaling pathways [89]. The MAPK and mTOR signaling pathways converge, and EIF4B is one of the focal points between both pathways, where survival and proliferation are increased. EIF4B has been shown to induce cell transformation, is considered an oncogene, and leads to further cancer risk [90]. Decreases in the phosphorylation of EIF4B lead to a reduction in viability, growth, and survival in TNBC [91]. Moreover, Toll/Interleukin-1-receptor domain-containing adaptor protein (TIRAP) plays a role in immune signaling and inflammatory signaling pathways. TIRAP activates NF-κB, MAPK3, and JNK signaling, resulting in the inflammatory response and cytokine release [92]. ETS-like (ELK) acts as an integration point for MAP kinase pathways and regulates a network of genes related to actin/migration [93]. The knockdown of ELK1 has reduced pancreatic cancer, breast cancer, and glioblastoma cells [94].

Fucoxanthin +TNF-α was found to downregulate the gene expression of RHOA, with an FC of −1.38, in TNF-α-stimulated MDA-MB-468 cells, and upregulate the expression of *RHOA*, with an FC of +1.89, in MDA-MB-231 cells in the PI3K-AKT signaling pathway. For the function of FAK-mediated cellular migration to occur, actin and cytoskeleton remodeling by small GTPases such as G protein Rho family protein A (RHOA) is required [95]. Guanine nucleotide exchange factor proteins facilitate converting the GDP-bound (inactive) to the GTP-bound (active) form, and GTPases facilitate converting from the active to the inactive form. RHOA GTPases are found to cycle between GDP and GTP-bound states and contribute to various processes such as cell polarity, cell morphogenesis, cell cycle progression, gene expression, vesicle trafficking, and the organization of microtubule and actin cytoskeletons [96].

In the present work, fucoxanthin was found to upregulate the expression of two genes, including *FOXO1* and *RASA1*, with FCs of +2.66 and +3.24, respectively, in TNF-α-stimulated MDA-MB-231 cells, and was found to upregulate EIF4EBP1, with an FC of +2.16, in TNF-α-stimulated MDA-MB-468 cells. Fucoxanthin downregulated the expression of EIF4EBP1, with an FC of −1.23, in MDA-MB-231, and downregulated the expression of two genes, including *FOXO1* and *RASA1*, with FCs of −1.21 and −1.19, respectively in TNF-α-stimulated MDA-MB-468, the PI3K-AKT signaling pathway of TNF-α-stimulated MDA-MB-231 cells. AKT has been found to inactivate tumor suppressor forkhead families of transcription factors such as FOXO1, which has been found to inactivate pro-caspase proteins such as Fas-L and Bad [97]. AKT directly phosphorylates FOXO, which promotes the nuclear elimination of FOXO-dependent transcription and suppresses FOXO activity [98]. The depletion of PI3K and activation of FOXO1 has led to cell cycle arrest and apoptosis in MCF-7 breast cancer cells [99]. 

Ras p21 protein activator 1 or Ras GTPase-activating protein (RASA1) expression is prevalent in TNBC tumors and is a tumor suppressor. RASA1 provides instruction for protein p120-RasGap and helps regulate Ras-MAPK [100]. Studies show that low expression of RASA1 is associated with TNBC and activates PI3K-AKT signaling. Loss of RASA1 expression is also involved in angiogenesis, metastasis, tumorigenesis, and invasion [101]. In breast cancer, EIF4EBP1 is highly overexpressed and frequently amplified, and is connected to increased metastasis, invasion, and tumor formation [102].

According to our findings, fucoxanthin has been shown to modulate several genes of the PI3K-AKT signaling pathway. These genes are involved in metastasis, invasion, cell growth, the cell cycle, proliferation, apoptosis inhibition, cytoskeleton and motility regulation, and microtubule and actin organization. To validate the results at the protein level, Wes analysis was performed. Fucoxanthin was shown to downregulate IRS-1, EIF4B, and ELK-1 and upregulate RHOA protein expression in TNF-α MDA-MB-231 cells. Fucoxanthin + TNF-α showed no change in the protein expression of EIF4B and ELK1 and upregulated RHOA protein expression in MDA-MB-468 cells. Low expression of this protein is known to initiate the actin cytoskeleton, permit cellular migration, encode small GTPases, and increase breast cancer metastasis. The cells’ actin cytoskeleton changes during mitosis, and actin cytoskeleton disruption leads to cell cycle arrest at the S phase [103]. These studies show that RHOA protein expression is associated with cell cycle arrest and cellular migration.

This study has certain limitations, one of which is the genetic variation between both cell lines. The molecular genetic profiles of Caucasian and African American TNBC cell line populations have not been well characterized [104]. Several studies have also revealed that the genomic profiles of TNBC cell lines vary, with altered gene expression observed in both Caucasian and African American TNBC cell lines [105].

As previously stated, several studies examined fucoxanthin’s effects on the PI3K-AKT signaling pathway in MDA-MB-231 cells; however, there was a lack of studies on MDA-MB-468 cells. The present study fills this gap, demonstrating that fucoxanthin modulated genes and proteins associated with the PI3K-AKT signaling pathway in both cell lines but showing different responses after fucoxanthin treatment. The PI3K-AKT signaling pathway is linked to cellular processes such as tumor cell migration, proliferation, angiogenesis, cell cycle progression, apoptosis, and cell adhesion. By showing the modulatory effects of fucoxanthin in genes and proteins of this pathway, this investigation indicates a possible molecular mechanism for fucoxanthin’s effects in both cell lines and shows fucoxanthin’s potential in halting cancer progression. 

## 4. Materials and Methods

### 4.1. Cell Lines, Chemicals, and Reagents

MDA-MB-231 (Caucasian American) and MDA-MB-468 (African American) TNBC cells were purchased from the American Type Culture Collection (ATCC). Heat-inactivated fetal bovine serum (HI-FBS), Dulbecco’s modified Eagle’s medium (DMEM) high glucose, penicillin/streptomycin, and phosphate-buffered saline (PBS) were obtained from Genesee Scientific (San Diego, CA, USA). Alamar Blue^®^, fucoxanthin (95% purity), dimethyl sulfoxide (DMSO), chloroform, and isopropyl alcohol were purchased from Sigma-Aldrich Co. (St. Louis, MO, USA). Human Angiogenesis Antibody Array (Cat# AAH-ANG-1000-4) and TNF-alpha (Cat# ELH-TNFα) were purchased from RayBiotech (Norcross, GA, USA). TRIzol was purchased from Thermo Fischer Scientific (Wilmington, DE, USA). PrimePCR_PI3K-AKT signaling pathway (SABTarget list) H96 arrays, SYBR Green, and iScript advanced reverse transcriptase kit were purchased from Bio-Rad (Hercules, CA, USA). A propidium iodide DNA staining kit was purchased from Abcam USA (Cambridge, MA, USA). The Wes kit and reagents used were obtained from ProteinSimple (San Jose, CA, USA). The antibodies used were obtained from Cell Signaling Technology (Danvers, MA, USA) and ThermoFisher (Waltham, MA, USA).

### 4.2. Cell Culture

TNBC cells (MDA-MB-231 and MDA-MB-468) were cultured in DMEM supplemented with 1% penicillin (100 U/mL)/streptomycin (0.1 mg/mL) and 10% heat-inactivated fetal bovine serum (HI-FBS). Cells were incubated at 5% CO_2_ and 37 °C, subcultured in T-75 flasks, and then, grown to 90% confluency before setting the cells for each assay. The plating used was composed of a DMEM experimental medium supplemented with 2.5% HI-FBS and no penicillin/streptomycin for each experiment.

### 4.3. Cell Viability 

Cells were seeded at a density of 3 × 10^4^ cells/100 µL/well in 96-well plates and incubated for attachment overnight in experimental media. After overnight incubation, cells were treated with control (DMSO + media), fucoxanthin (6.25 µM), TNF-α (50 ng/mL), and fucoxanthin (6.25 µM) + TNF-α (50 ng/mL) and incubated for another 24 h. In our previous study, we tested various concentrations of fucoxanthin (1.56–300 μM) alone at 24 h (Ahmed et al., 2023). In the current study, we selected fucoxanthin (6.25 μM) at 24 h because this concentration shows cell viability at more than 75% in both cell lines. The compound fucoxanthin was dissolved in DMSO and then in a medium, with the final concentration of DMSO not exceeding 0.1%. A volume of 100 µL of each treatment was added to the plates containing cells, with a final volume of 200 µL. Alamar Blue^®^ (Resazurin) assay was used to assess cell viability, and 20 µL of Alamar Blue^®^ solution (0.5 mg/mL) was added to each plate and incubated for a total of 4 h. An Infinite M200 microplate reader (Tecan Trading AG) was used to read the fluorescence of excitation/emission at 550/580 nm wavelengths for quantification. Fluorescence changes were observed as resazurin was reduced to resorufin by the viable cells. The fluorescent signal was proportional to the number of viable cells in the sample, and the data were displayed as a percentage of live, untreated controls.

### 4.4. cDNA Synthesis and RT-PCR Array (PI3K-AKT Signaling Pathway)

#### 4.4.1. Extraction of RNA

Cells were exposed to different treatments for 24 h, and treatments included control (cells + DMSO), fucoxanthin treatment (6.25 µM), TNF-α stimulation (50 ng/mL), and co-treatment with fucoxanthin (6.25 µM) + TNF-α (50 ng/mL). After 24 h, cells were harvested, and cell pellets were collected. An amount of 1 mL of TRIzol was added to each homogenized pellet. Next, 200 μL of chloroform was added to each lysed sample. For 3–5 min, the samples sat at room temperature on a rack, were vortexed for 15–30 s, and then centrifuged at 11,000 rpm for 12 min at 2–8 °C. New tubes containing 500 μL of isopropyl alcohol were used to collect the supernatant of each sample. Tubes were inverted 3 times and, for 10 min, were incubated at room temperature. For RNA precipitation, samples were centrifuged at 11,000 rpm for 12 min at 2–8 °C, and the sample supernatant was removed. Pellets were washed with 75% ethanol (inverted carefully) and centrifuged at 9000 rpm for 5 min at 2–8 °C. The ethanol was aspirated, and for 15 min, tubes sat at room temperature until ethanol droplets were evaporated. The pellets were dissolved in 35 μL of RNase-free water and incubated for 30 min on ice. The NanoDrop Spectrophotometer (Thermo Fischer Scientific, Wilmington, DE, USA) was used for each sample to determine RNA purity and quantification.

#### 4.4.2. cDNA Conversion and RT-PCR

The cDNA strands were synthesized from mRNA using iScript advanced transcriptase from Bio-Rad. A combination of 4 µL of the 5X iScript advanced reaction mix, 1 µL of reverse transcriptase, 7.5 µL of the sample (1.5 µg/reaction), and 7.5 µL of water was combined in a 0.2 mL tube to make a total volume of 20 µL. Reverse transcription was performed using the thermocycler program, and the transcription steps included 46 °C for 20 min and 95 °C for 1 min. Next, for RT-PCR array studies, a combination of 5 µL of the sample (200 ng cDNA/reaction) and 1045 µL of nuclease-free water was mixed and vortexed to make a cDNA dilution. Then, 30 µL from the cDNA dilution and 3 µL of the PCR control assay were mixed and vortexed in a small tube to prepare the PCR control and added to the positive control well. Then, 10 µL of the cDNA dilution and 10 µL of SYBR Green were combined and added to each well except the control well. For the positive control well, a combination of 10 µL of the PCR control assay dilution and 10 µL of SYBR Green were combined. RT-PCR was performed at 95 °C for 2 min, and denaturation was carried out at 95 °C for 10 s, followed by 39 cycles of 60 °C for 30 s (annealing/extension), and 65–95 °C for 5 s/step (Melting curve) using the Bio-Rad CFX96 Real-Time System (Hercules, CA, USA). The PCR arrays (PI3K-AKT signaling pathway SAB Target List H96—Bio-Rad Hercules, CA, USA) were specific to genes that are involved in the PI3K-AKT signaling pathway.

### 4.5. Wes Analysis

A Wes machine (ProteinSimple, San Jose, CA, USA) was utilized to determine total proteins. ProteinSimple provided reagents, and analysis was performed following the user manual. Protocols were adjusted for each antibody and protein loading. Cells were exposed to the following treatments: control (media + DMSO), fucoxanthin (6.25 µM), TNF-α (50 ng/mL), and a combination of fucoxanthin (6.25 µM) + TNF-α (50 ng/mL). After a 24-h treatment, cells were washed and pelleted, and a lysis buffer comprising a protease inhibitor cocktail was added to each pellet. Protein concentrations of 0.25 mg/mL, 0.50 mg/mL, and 1 mg/mL were used. Proteins were integrated with a master mix to obtain final concentrations of 0.25 mg/mL to 1 mg/mL total protein, 1x sample buffer, 1x fluorescent molecular weight markers, and 40 mM dithiothreitol. For 5 min, samples were heated to 95 °C. Blocking solution, samples, primary antibodies (dilution: 1:5 to 1:125), a chemiluminescent substrate, horseradish peroxidase-conjugated secondary antibodies, and separation and stacking matrices were inserted into assigned wells on a microplate. Target protein identification was analyzed using a primary antibody specific to each protein, an HRP-conjugated secondary antibody, and a chemiluminescent substrate. The microplate was loaded and inserted in the instrument where fully automated electrophoresis and immunodetection occurred following the manufacturer’s protocol (ProteinSimple). The reaction occurred within the capillary system utilizing exclusive antibodies; the proteins were detected, the chemiluminescence reaction was elucidated, and digital blot images were captured. A charge-coupled-device camera captured chemiluminescence, and the digital image was quantified and analyzed using ProteinSimple Compass software (Wes-WS2709). Protein expression was normalized using beta-actin as the loading control, and normalization was also calculated using ProteinSimple Compass Software. The selected antibodies specific to each protein from Cell Signaling Technology^®^ were as follows: IRS-1 (59G8) Rabbit mAb #2390S, EIF4B Antibody #3592S, RhoA (67B9) Rabbit mAb #2117S, Elk-1 Antibody #9182S, and Beta-Actin (13E5) Rabbit mAb #4970S.

### 4.6. Data Analysis

Statistical analysis was performed using GraphPad Prism (version 6.07). All data were expressed as mean ± SEM from at least 3 independent experiments, and the significance of the difference between the groups was assessed using Student’s *t*-test and a one-way ANOVA, followed by Dunnett’s multiple comparisons test. * *p* < 0.05, ** *p* < 0.01, *** *p* < 0.001, and **** *p* < 0.0001, and ns = *p* > 0.05. KEGG (Kyoto Encyclopedia of Genes and Genomes: https://www.genome.jp/kegg/ accessed on 12 June 2023) and STRING (Search Tool for Retrieving Interacting Genes/proteins: https://string-db.org/ accessed on 15 June 2023) databases were utilized to display gene, protein, and signaling pathway interactions.

## 5. Conclusions

**Figure 13:**
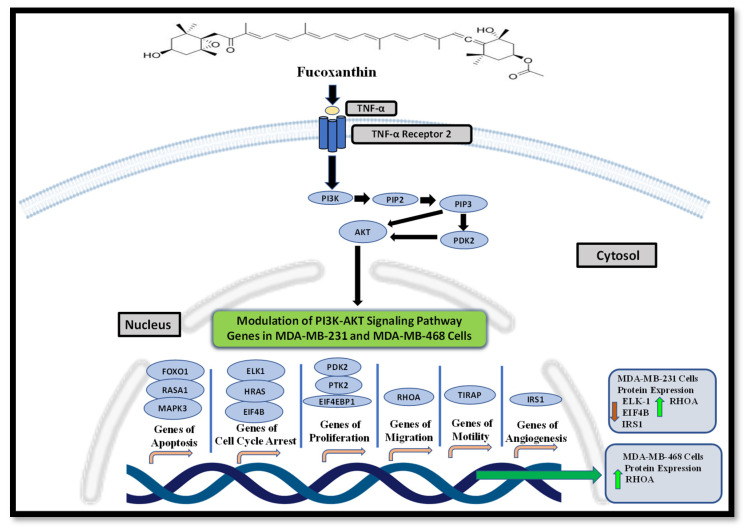
Proposed molecular effects of fucoxanthin through modulation of genes and proteins of the PI3K-AKT signaling pathway in TNF-α-stimulated MDA-MB-231 and MDA-MB-468 cells. The black arrow shows that fucoxanthin modulated the PI3K-AKT signaling pathway genes in MDA-MB-231 and MDA-MB-468 cells. Red arrows indicate inhibition by fucoxanthin, and green arrows show activation by fucoxanthin. The graph was created utilizing Microsoft PowerPoint version 1808.

In conclusion, fucoxanthin modulated several genes of the PI3K-AKT signaling pathway that are differentially expressed in each of the MDA-MB-231 and MDA-MB-468 cell lines. The differential expression of the identified genes supports the abnormal activation of the signaling pathway that leads to mitotic spindle formation, a dysregulated cell cycle, continued division, and angiogenesis. Fucoxanthin has been shown to be effective at modulating several genes and proteins in the PI3K-AKT signaling pathway that aid in inducing cell cycle arrest and apoptosis or inhibiting mitotic spindle formation and cellular division (Figure 13), indicating that this pathway may be the molecular signaling involved in fucoxanthin’s anticancer effects. This study shows that fucoxanthin may be a promising chemotherapeutic for breast cancer therapy and could be combined with chemotherapy to enhance synergistic therapeutic efficacy in TNBC.

## Figures and Tables

**Table 1:** Data show FCs for 3 genes that were significantly upregulated in MDA-MB-231 cells, which include *FOXO1* (+2.66), *RASA1* (+3.24), and *RHOA* (+1.89), and FCs for 10 genes that were significantly downregulated in MDA-MB-231 cells, which include *HRAS* (−1.97), *MAPK3* (−1.90), *PDK2* (−2.46), *IRS1* (−3.51), *EIF4EBP1* (−1.23), *EIF4B* (−3.83), *PTK2* (−2.64), *TIRAP* (−2.14), and *ELK1* (−3.91).

Gene Name	Description	Fold Change (Downregulated or Upregulated)	Function
*RASA1*	Ras P21 Protein Activator 1	+3.24	Provides instruction for protein p120-RasGap and helps regulate Ras-MAPK [35].
*FOXO1*	Forkhead Box 1	+2.66	Tumor suppressor that induces cell cycle arrest and apoptosis [36].
*RHOA*	Rho Family Protein A	+1.89	Contributes to cell cycle progression, adhesion, and microtubule and actin cytoskeleton organization [37].
*ELK1*	ETS Transcription Factor 1	−3.91	Regulates a network of genes related to actin/migration [38].
*EIF4B*	Eukaryotic Translation Initiation Factor 4B	−3.83	Regulated by both Ras-MAPK and PI3K/mTOR and plays a role in cell viability, cell growth, and survival [39].
*IRS1*	Insulin Receptor Substrate 1	−3.51	Regulates cell motility, proliferation, and cell survival [40].
*PTK2*	Protein Tyrosine Kinase 2	−2.64	Stimulates tumor cell invasiveness and adhesion [41].
*PDK2*	Pyruvate Dehydrogenase Kinase 2	−2.46	Associated with proliferation, anti-apoptosis, tumor aggressiveness, and therapy resistance [42].
*TIRAP*	TIR Domain-Containing Adaptor Protein	−2.14	Activates NF-κB, MAPK3, and JNK signaling, resulting in the inflammatory response and cytokine release [43].
*HRAS*	HRas Proto-Oncogene, GTPase	−1.97	Regulates adhesion, cytoskeleton rearrangements, proliferation, cell survival, and cell motility [44].
*MAPK3*	Mitogen-Activated Protein Kinase 3	−1.90	Regulates inflammation, proliferation, apoptosis, and differentiation [45].
*EIF4EBP1*	Eukaryotic Transition Initiation Factor 4E-Binding Protein 1	−1.23	Increases metastasis, invasion, and tumor formation [46].

**Table 2:** Data show FC for 1 gene significantly upregulated in MDA-MB-468 cells: *EIF4EBP1* (+2.16). Also shown are FCs for 11 genes that were significantly downregulated in MDA-MB-468 cells, which include *FOXO1* (−1.21), *HRAS* (−1.34), *MAPK3* (−1.22), *PDK2* (−1.14), *RASA1* (−1.19), *IRS1* (−1.49), *EIF4B* (−1.51), *PTK2* (−1.51), *TIRAP* (−1.61), *RHOA* (−1.38), and *ELK1* (−1.44).

Gene Name	Description	Fold Change (Downregulated or Upregulated)	Function
*EIF4EBP1*	Eukaryotic Transition Initiation Factor 4E-Binding Protein 1	+2.16	Increases metastasis, invasion, and tumor formation [46].
*TIRAP*	TIR Domain-Containing Adaptor Protein	−1.61	Activates NF-κB, MAPK3, and JNK signaling, resulting in the inflammatory response and cytokine release [43].
*EIF4B*	Eukaryotic Translation Initiation Factor 4B	−1.51	Regulated by both Ras-MAPK and PI3K/mTOR, it plays a role in cell viability, growth, and survival [39].
*PTK2*	Protein Tyrosine Kinase 2	−1.51	Stimulates tumor cell invasiveness and adhesion [41].
*IRS1*	Insulin Receptor Substrate 1	−1.49	Regulates cell motility, proliferation, and cell survival [40].
*ELK1*	ETS Transcription Factor 1	−1.44	Regulates a network of genes related to actin/migration [38].
*RHOA*	Rho Family Protein A	−1.38	Contributes to cell cycle progression, adhesion, and microtubule and actin cytoskeleton organization [37].
*HRAS*	HRas Proto-Oncogene, GTPase	−1.34	Regulates adhesion, cytoskeleton rearrangements, proliferation, cell survival, and cell motility [44].
*MAPK3*	Mitogen-Activated Protein Kinase 3	−1.22	Regulates inflammation, proliferation, apoptosis, and differentiation [45].
*FOXO1*	Forkhead Box 1	−1.21	Tumor suppressor that induces cell cycle arrest and apoptosis [36].
*RASA1*	Ras P21 Protein Activator 1	−1.19	Provides instruction for protein p120-RasGap and helps regulate Ras-MAPK [35].
*PDK2*	Pyruvate Dehydrogenase Kinase 2	−1.14	Associated with proliferation, anti-apoptosis, tumor aggressiveness, and therapy resistance [42].

**Table 3:** This table displays interaction scores of proteins in the PI3K-AKT signaling pathway.

Node 1	Node 2	Node l Accession	Node 2 Accession	Score
RHOA	PDK2	ENSPO0000400175	ENSP00000420927	0.184
RHOA	IRS1	ENSPO0000400175	ENSP0000030489S	0.386
RHOA	FOXO1	ENSP00000400175	ENSP00000368880	0.364
RHOA	EIF4B	ENSP00000400175	ENSP00000388806	0.324
PDK2	RHOA	ENSP00000420927	ENSPO0000400175	0.184
PDK2	IRS1	ENSPO0000420927	ENSPO0000304895	0.436
PDK2	FOXO1	ENSP00000420927	ENSP00000368880	0.413
PDK2	EIF4B	ENSP00000420927	ENSP00000388806	0.179
IRS1	RHOA	ENSP00000304895	ENSPO0000400175	0.386
IRS1	PDK2	ENSP00000304895	ENSPO0000420927	0.436
IRS1	FOXO1	ENSP00000304895	ENSP00000368880	0.854
IRS1	EIF4B	ENSPO0000304895	ENSP00000388806	0.357
FOXO1	RHOA	ENSP00000368880	ENSPO0000400175	0.364
FOXO1	PDK2	ENSP00000368880	ENSP00000420927	0.413
FOXO1	IRS1	ENSP00000368880	ENSP00000304895	0.854
FOXO1	EIF4B	ENSP00000368880	ENSPO0000388806	0.244
EIF4B	RHOA	ENSP00000388806	ENSP00000400175	0.324
EIF4B	PDK2	ENSPO0000388806	ENSPO0000420927	0.179
EIF4B	IRS1	ENSPO0000388806	ENSPO0000304895	0.357
EIF4B	FOXO1	ENSPO0000388806	ENSPO0000368880	0.244

## Data Availability

All data from this study are included in this published article.
